# Endoplasmic reticulum stress in chondrodysplasias caused by mutations in collagen types II and X

**DOI:** 10.1007/s12192-016-0719-z

**Published:** 2016-08-15

**Authors:** Katarzyna Gawron

**Affiliations:** Microbiology Department, Faculty of Biochemistry, Biophysics and Biotechnology, Jagiellonian University, Gronostajowa 7, 30-387 Krakow, Poland

**Keywords:** Endoplasmic reticulum stress, Unfolded protein response, Mutation, Collagen, Chondrodysplasia, Mechanism

## Abstract

The endoplasmic reticulum is primarily recognized as the site of synthesis and folding of secreted, membrane-bound, and some organelle-targeted proteins. An imbalance between the load of unfolded proteins and the processing capacity in endoplasmic reticulum leads to the accumulation of unfolded or misfolded proteins and endoplasmic reticulum stress, which is a hallmark of a number of storage diseases, including neurodegenerative diseases, a number of metabolic diseases, and cancer. Moreover, its contribution as a novel mechanistic paradigm in genetic skeletal diseases associated with abnormalities of the growth plates and dwarfism is considered. In this review, I discuss the mechanistic significance of endoplasmic reticulum stress, abnormal folding, and intracellular retention of mutant collagen types II and X in certain variants of skeletal chondrodysplasia.

## Introduction

The endoplasmic reticulum (ER) is an eukaryotic organelle responsible for synthesis, folding, trafficking, and sorting of proteins. It also plays an important role in lipid and steroid synthesis as well as calcium homeostasis (Engin and Hotamisligil 2010; Hosoi and Ozawa 2009). An imbalance between the load of unfolded proteins and the processing capacity in ER leads to the accumulation of unfolded or misfolded proteins and ER stress (Ron and Walter 2007; Schröder and Kaufman 2005; Sano and Reed 2013). To alleviate accumulation of unfolded proteins, the process being toxic to cells, ER triggers an evolutionarily conserved signaling cascade, named unfolded protein response (UPR) (Engin and Hotamisligil 2010; Schröder and Kaufman 2005; Tsuru et al. 2016; Lin et al. 2008). Adaptation and restoration of ER function through UPR signaling comprise translational attenuation of global protein synthesis, upregulation of ER chaperones and prevention from trafficking (to the proper subcellular localizations), as well as degradation of unstable or partially folded mutant proteins by an endoplasmic reticulum-associated degradation (ERAD) system (Engin and Hotamisligil 2010; Hosoi and Ozawa 2009; Ron and Walter 2007; Schröder and Kaufman 2005; Bernales et al. 2006). If ER stress is excessive or prolonged and cannot be resolved, signaling switches from prosurvival to proapoptotic (Hosoi and Ozawa 2009; Szegezdi et al. 2006). ER stress and UPR are critical for the normal cellular homeostasis and development of the organism and are also known to play major roles in the pathogenesis of many diseases such as neurodegenerative (Uehara et al. 2006; Hoozemans et al. 2007; Perri et al. 2016) and cardiovascular (Minamino et al. 2010; Kassan et al. 2012) diseases, diabetes (Basha et al. 2012), obesity (Hosoi et al. 2008), inflammation (Ha et al. 2015), and cancer (Suh et al. 2012; Chhabra et al. 2015; Lin et al. 2008; Mahdi et al. 2016; Yoshida 2007a). In recent years, accumulating evidence indicates that abnormal folding and accumulation of structural proteins that are produced to fulfil their physiological function in the extracellular space of tissues may lead to ER stress and induction of a wide range of systemic diseases. For instance, several reports linked ER stress to mutations in collagen type II that induce various forms of spondyloepiphyseal dysplasia (SED) (Chung et al. 2009; Okada et al. 2015; Liang et al. 2014). Other studies highlight the role of ER stress in the pathogenesis of metaphyseal (chondro) dysplasia, Schmid type (MCDS), a disorder caused by mutations in the collagen type X, characterized by expansion of the hypertrophic zone of the growth plate resulting in dwarfism (Rajpar et al. 2009; Ho et al. 2007; Mäkitie et al. 2010). Another studies point out the crucial role of ER stress in diseases caused by mutations in collagen type VI genes that lead to the mild to severe phenotype of myopathies, including Ullrich congenital muscular dystrophy (UCMD) and Bethlem myopathy (BM) (De Palma et al. 2014), or mutations in collagen type IV genes (COL4A3/COL4A4/COL4A5) that contribute to the pathogenesis of thin-basement-membrane nephropathy (TBMN) and Alport syndrome (AS) (Pieri et al. 2014) and cataract development (Firtina et al. 2009). Succeeding examples comprise ocular dysgenesis, myopathy, brain malformations, cerebrovascular disease, and cerebral hemorrhages, all caused by mutations in collagen type IV (COL4A1/COL4A2) (Kuo et al. 2014; Jeanne et al. 2012; Verbeek et al. 2012; Volonghi et al. 2010; Gould et al. 2007). Moreover, accumulating evidence shows an association between mutations in genes encoding proteins involved in type I procollagen processing or chaperoning and pathogenesis of recessive variants of osteogenesis imperfecta (OI), while dysregulation of molecular chaperones is implicated in ER stress in severe forms of osteoarthritis (OA) (Lisse et al. 2008; Kelley et al. 2011; Marini et al. 2010; Bodian et al. 2009; Symoens et al. 2013; Nugent et al. 2009; Takada et al. 2011).

In this review, basic mechanisms of ER stress and its significance in certain variants of chondrodysplasia caused by mutations in collagen types II and X are discussed.

## Endoplasmic reticulum stress

**Fig. 1:**
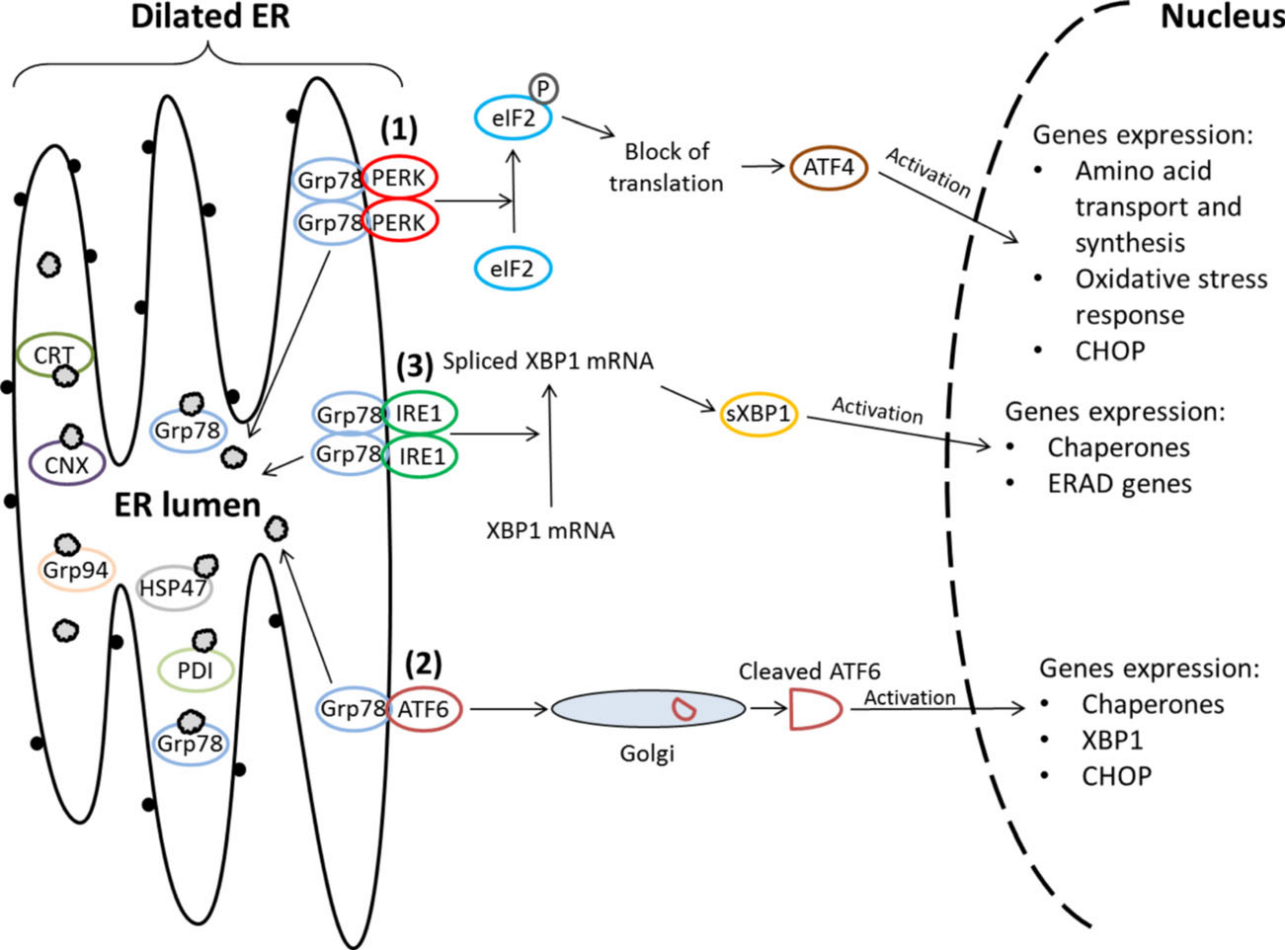
Endoplasmic reticulum signaling triggers unfolded protein response. Under physiological conditions, glucose-regulated protein 78 kDa (Grp78/BiP), protein disulfide isomerase (PDI), heat shock protein 47 (HSP47), and other molecular chaperones are present in the lumen of endoplasmic reticulum (ER). Additionally, Grp78 binds the ER luminal domains of the three ER stress receptors, *i.e.,* protein kinase RNA-like endoplasmic reticulum kinase (PERK), activating transcription factor 6 (ATF6), and inositol-requiring enzyme 1 (IRE1). Accumulation of unfolded proteins in the ER, *e.g.,* as a result of mutation in the collagen gene, induces sequential dissociation of Grp78 from PERK (*1*), ATF6 (*2*), and IRE1 (*3*), respectively, and their activation. Dissociated Grp78 molecules and other chaperones are mobilized to form complexes with unfolded proteins aggregated in the ER. Activated PERK (via dimerization and autophosphorylation) phosphorylates eukaryotic initiation factor 2α (eIF2α). This phosphorylation suppresses general protein synthesis, thus decreasing the entry of newly synthesized proteins into the ER and enabling translation of ATF4. ATF4 translocates to the nucleus and induces the transcription of genes required to restore ER homeostasis including that for CCAAT/enhancer binding protein homologous protein (CHOP). ATF6 is activated by limited proteolysis after its translocation from the ER to the Golgi apparatus. It is cleaved by site 1 and site 2 proteases (S1P, S2P) releasing the cytoplasmic 50kDa domain (ATF6_50_) which is an active transcription factor. ATF6_50_ regulates the expression of genes involved in the unfolded protein response (UPR), including chaperones, CHOP, and X-box binding protein 1 (XBP1) . Additionally, the activation of XBP1 is carried out by IRE1. Activated IRE1 produces an unconventional splice in cytoplasmic XBP1 mRNA. Spliced XBP1 protein (sXBP1) translocates to the nucleus and upregulates the transcription of genes encoding chaperones to increase the protein folding capacity of the ER and genes controlling the endoplasmic reticulum-associated degradation (ERAD) system, a mechanism by which misfolded protein is retrotranslocated into the cytoplasm and degraded in the proteasome. This complex action aims to restore ER homeostasis by blocking unfolded protein aggregation, inducing degradation of aggregated proteins and enhancing folding capacity

The endoplasmic reticulum is a membranous organelle and is one of the major compartments for the biosynthesis, maturation, and folding of proteins. The specialized intra-ER environment contains several factors that are required for the formation of disulfide bonds and for optimal protein folding, including ATP, Ca^2+^, and unique oxidizing conditions. The accuracy of protein folding is also monitored by Ca^2+^-dependent molecular chaperones, such as glucose-regulated protein 78 kDa (Grp78/BiP), glucose-regulated protein 94 kDa (Grp94), calreticulin (CRT), and calnexin (CNX) which stabilize the protein folding intermediates and prevent the aggregation of proteins in the ER (Szegezdi et al. 2006; Anelli and Sitia 2008; Kim et al. 2008; Hetz 2012; Mahdi et al. 2016). The physiological state of the ER is challenged when the influx of the nascent unfolded or misfolded polypeptides exceeds the processing capacity of the ER (Schröder and Kaufman 2005; Sano and Reed 2013). Correct protein folding may fail as a result of gene mutations, RNA modifications, amino acid misincorporation during translation, or unequal synthesis of individual subunits of multisubunit protein species. Furthermore, posttranslational modifications can be caused by changes in pH, temperature, ionic strength, and oxidative and other stresses (Kim et al. 2008; Hetz 2012; Schröder 2008). The unfolded/misfolded proteins expose hydrophobic domains that are normally hidden within the correctly folded polypeptide’s structure. The interaction between such inappropriately folded proteins may lead to their accumulation within the cells. In turn, aggregates may interfere with cellular functions and are potentially cytotoxic. Three hypotheses have been proposed to explain cytotoxic mechanisms: (i) the channel hypothesis suggesting that ring-shaped oligomers form pores in cellular membranes, (ii) misfolded proteins lead to dysfunction of normal proteins with which they interact, and (iii) misfolding proteins may deplete components of the quality control system, which consists of chaperone and degradation systems, thereby reducing their functionality (Barral et al. 2004). To restore cellular integrity and homeostasis, cells activate evolutionarily conserved adaptive response, called UPR, that consists of several quality control systems (Schröder and Kaufman 2005; Lin et al. 2008; Szegezdi et al. 2006; Schröder 2008) that act in concert for (i) inhibiting general protein translation, (ii) activating the signaling pathways that lead to expression of molecular chaperones and increasing folding capacity, and (iii) promoting the degradation of misfolded proteins and reducing aggregation (Engin and Hotamisligil 2010; Anelli and Sitia 2008; Kim et al. 2008; Hetz 2012). UPR is mediated through activation of three ER transmembrane receptors: inositol-requiring enzyme 1 (IRE1), activating transcription factor 6 (ATF6), and protein kinase RNA-like endoplasmic reticulum kinase (PERK). Under physiological conditions, all three factors related to ER stress are maintained in inactive form through their association with Grp78 (Anelli and Sitia 2008). Upon accumulation of unfolded proteins in the lumen of the ER, Grp78 dissociates from IRE1, ATF6, and PERK, which leads to their activation and assistance in folding processes (Szegezdi et al. 2006; Anelli and Sitia 2008; Shen et al. 2002; Rutkowski and Kaufman 2004). PERK is a type 1 transmembrane protein kinase residing in the ER which is activated in response to perturbation of protein folding and constitutes a signal transducer of the earliest phase of stress response (Liu et al. 2016). UPR first alleviates the ER stress by diminishing the overall protein load via PERK-mediated phosphorylation of the α subunit of eukaryotic translation initiation factor 2 (eIF2α) (Mahdi et al. 2016; Liu et al. 2016; Joshi et al. 2013; Zhang et al. 2013). In turn, the decrease in eIF2 activity promotes the translation of activating transcription factor 4 (ATF4) resulting in the activation of CCAAT/enhancer binding protein (C/EBP) homologous protein (CHOP) production, which promotes ER stress-induced apoptosis (Ron and Walter 2007; Walter and Ron 2011; Fawcett et al. 1999; Jiang et al. 2004). ATF6 is a basic leucine zipper (bZIP) transcription factor that upon dissociation of Grp78 translocates to the Golgi complex, in which it undergoes proteolytic cleavage by the serine protease (S1P) and the metalloprotease (S2P) (sites 1 and 2, respectively) (Shen et al. 2002; Zhang et al. 2013; Nadanaka et al. 2006). The cleaved 50 kDa cytoplasmic bZIP-containing fragment translocates to the nucleus, where it binds to the ER stress response element (ERSE) and activates genes involved in the adaptive stress response, *i.e.,* Grp78, CHOP, and X-box binding protein 1 (XBP1) (Anelli and Sitia 2008; Nadanaka et al. 2006; Brown et al. 2000). It has been shown that cell- or tissue-specific UPR may exist under ER stress; for example, cAMP response element binding protein H (CREBH) has been identified as a regulated intramembrane proteolysis (RIP)-regulated liver-specific transcription factor that is cleaved upon ER stress (Zhang et al. 2006) while old astrocyte specifically induced substance (OASIS), which is similar to the ATF6 family, has been identified as a transducer of ER stress in astrocytes (Kondo et al. 2005). IRE1 is a type 1 transmembrane serine/threonine receptor protein kinase which is activated via homodimerization and trans-autophosphorylation upon dissociation from Grp78 (Hosoi and Ozawa 2009; Zhang et al. 2013). The activated domain of IRE1 acts like a nuclease and catalyzes the removal of a small intron from XBP1 messenger RNA (mRNA) (Zhang et al. 2013; Yoshida 2007b). This splicing creates a translational frameshift in XBP1 to produce an active transcription factor which directly binds to the ERSE and the UPR element (UPRE), leading to the upregulation of genes such as ER chaperones, *e.g.,* Grp78 gene (*HSPA5*) (Hosoi and Ozawa 2009; Suh et al. 2012; Anelli and Sitia 2008; Shen et al. 2002) (Fig. 1).

**Fig. 2:**
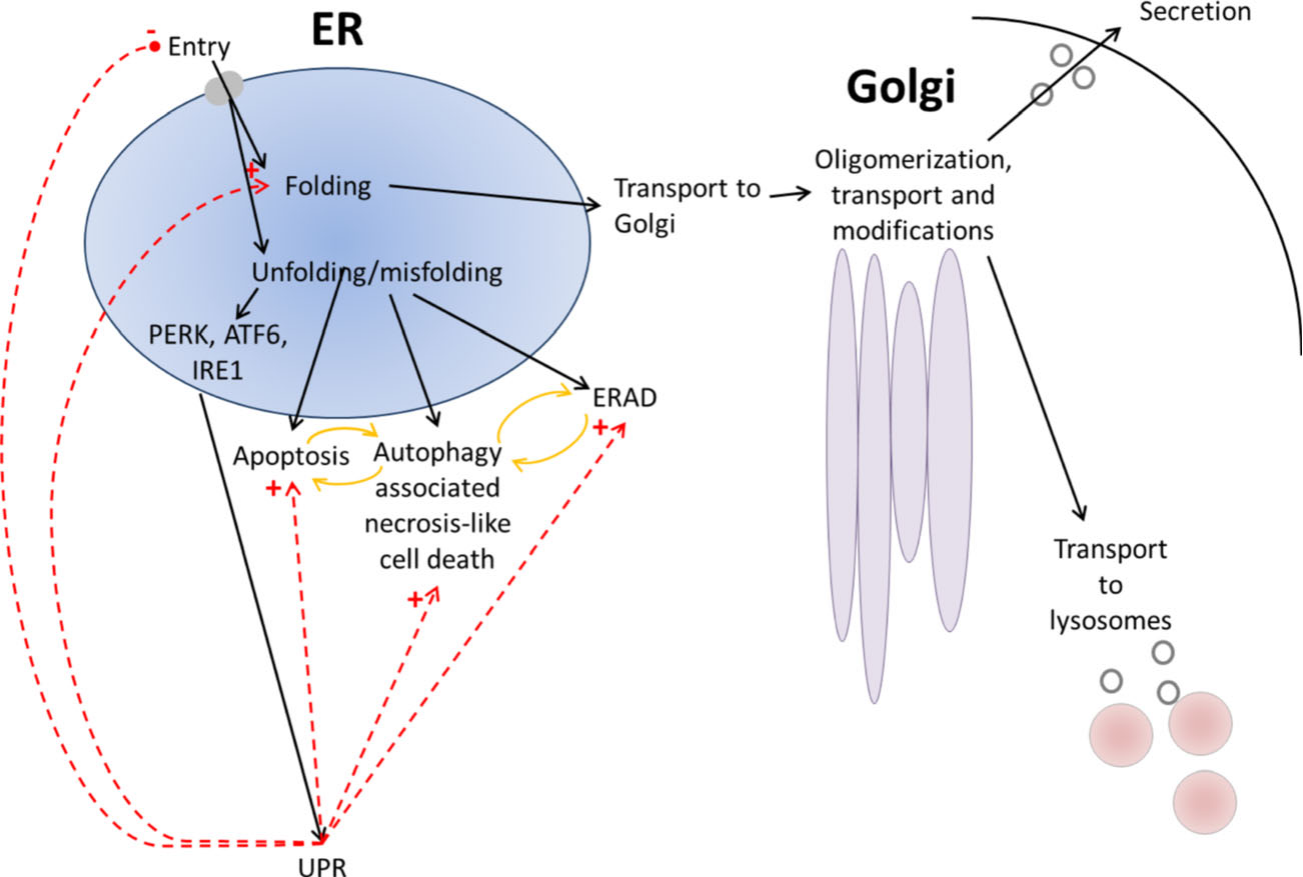
Quality control interplay in the “ER-Golgi-lysosomes/extracellular space axis.” Extracellular space proteins, *e.g.,* collagens, are synthesized by ribosomes and translocated into the endoplasmic reticulum (ER). In the ER, proteins accomplish their native form (folding, assembling) under strict quality control mechanisms. Appropriate folding/structure of the proteins enables their transport and modification in the Golgi, followed by transport to the extracellular space and/or to lysosomes. Accelerated aggregation of unfolded/misfolded proteins which overload the folding capacity within the ER induces the ER stress sensors, *i.e.*, protein kinase RNA-like endoplasmic reticulum kinase (PERK), activating transcription factor 6 (ATF6), and inositol-requiring enzyme 1 (IRE1) which further activate the unfolded protein response (UPR) signaling. Unfolded/misfolded proteins are retained and directed to degradation by endoplasmic reticulum-associated degradation (ERAD) system, apoptosis, or autophagy (reciprocal regulation, *yellow arrows*). Under the quality control mechanisms, the ER stress aims to restore the homeostasis within ER through regulation of protein entry into the ER, folding, and degradation. *Black arrows* show the alternative pathways of the ER-Golgi-lysosomes/extracellular space axis depending on the proper folding, unfolding, or misfolding of protein. *Red, intermittent arrows* depict homeostatic control pathways (*plus sign* stimulatory, *minus sign* inhibitory)

Protein quality control primarily promotes cell survival and the maintenance of cellular homeostasis by switching on several pathways of the adaptive response. One of the central ER stress defense strategies is to upregulate coordinately its chaperoning capacity. Chaperones are specialized proteins that play a key role in cellular homeostasis by assisting in protein folding, assembly of the macromolecular complexes, protein transport, and cellular signaling (Ullman et al. 2008). In the ER, the main function of chaperones is to prevent inappropriate aggregation of nascent peptide chains during protein synthesis. Chaperones bind to and stabilize exposed hydrophobic domains of target proteins and promote proper folding of newly synthesized proteins. This process involves cycles of controlled binding and release of target polypeptides (Horwich 2004; Chaudhuri and Paul 2006). While some chaperones non-specifically interact with a wide variety of polypeptides, others are restricted to specific subsets or even individual proteins. Chaperones are expressed and maintained at steady levels in unstressed cell; however, their expression is highly upregulated during stress conditions. Dysregulated chaperonic activity results in unfolded, misfolded, or aggregated proteins that will eventually be targeted to degradation pathways or accumulate in cells, leading to impairment of function and eventually contributing to various diseases. Apart from their critical role in protein folding, chaperones also function as signal-transducing molecules by affecting the transition between active and inactive states of signaling molecules, changing their subcellular localization or affecting protein-protein interactions (Engin and Hotamisligil 2010). Interestingly, chaperones are able to distinguish between the native and non-native states of targeted proteins, but how they discriminate between correctly and incorrectly folded proteins and how they target the latter for degradation are yet to be explored (Vembar and Brodsky 2008). Apart from molecular chaperones, the ubiquitin proteasome pathway (UPP) is referred to as the second part of a protein quality control system (PQCS) and plays a critical role in cell function and survival. It has been shown that disturbance in or impairment of the UPP, which may be induced by the accumulation of misfolded proteins in the ER or loss of function of the enzymes involved in the ubiquitin conjugation and deconjugation pathway, leads to altered UPP function, which positively affects the accumulation of protein aggregates in the cell. The formation of oligomers and aggregates occurs in the cell when a critical concentration of misfolded protein is reached. Aggregated proteins inside the cell often lead to the formation of “an amyloid-like structure,” which eventually causes different types of degenerative disorders and, ultimately, cell death (Anelli and Sitia 2008). Finally, when correct folding is impossible and degradation by the UPP is insufficient, then the UPR switches from being prosurvival to proapoptotic (Engin and Hotamisligil 2010; Schröder and Kaufman 2005). Typically, excessive ER stress leads to apoptosis via an increase in the expression of CHOP or by inducing caspase-dependent apoptosis (Lin et al. 2008; Szegezdi et al. 2006; Chiribau et al. 2010). In certain circumstances when excessive ER stress fails to induce apoptosis, autophagy might also serve as an alternative route leading to cell death and necrosis (Szegezdi et al. 2006; Suh et al. 2012; Ullman et al. 2008) (Fig. 2).

## Endoplasmic reticulum stress and the diseases

Many inherited disorders of connective tissue are caused by mutations in genes encoding structural components of the extracellular matrix (ECM) or enzymes that participate in their posttranslational modifications and assembly. Often the mutations interfere with ECM protein folding and inhibit their secretion from the cell resulting in disruption of their function in connective tissue. Therefore, although the prevailing paradigm for inherited diseases of the ECM has involved predominantly extracellular molecular pathology, more recently, an increasing number of reports have shown that intracellular processes may affect the pathology of these conditions as well (Bateman et al. 2009).

Up to now, the role of ER stress and the activation of pathways leading to UPR have been intensively investigated in a number of neurodegenerative disorders, *e.g.,* Alzheimer’s and Parkinson’s diseases (Perri et al. 2016; Uehara et al. 2006; Hoozemans et al. 2007; Ron et al. 2010), cancer (Chhabra et al. 2015; Suh et al. 2012; Ha et al. 2015), and chronic metabolic diseases such as obesity, insulin resistance, and type 2 diabetes (Hotamisligil 2010); in a number of lysosomal storage disorders, *e.g.*, Fabry’s and Gaucher’s diseases (Yam et al. 2006; Maor et al. 2013; Ron et al. 2010); and in the pathology of diseases involving professional secretory tissues such as cystic fibrosis and α1-antitrypsin deficiency (Bartoszewski et al. 2011; Alam et al. 2014). Recent reports investigating effects of mutations on assembly and secretion of several ECM components suggest that multiple outcomes such as misfolding and intracellular accumulation of mutant ECM proteins may result from induction of ER stress (Chung et al. 2009; Liang et al. 2014; Rajpar et al. 2009; Pieri et al. 2014; Firtina et al. 2009; Gould et al. 2007; Nugent et al. 2009; Bateman et al. 2009; Boot-Handford and Briggs 2010; Yang et al. 2005).

## Mutations leading to cartilage pathology

Cartilage is a connective tissue that serves multiple functions during embryonic development and in postnatal life. It is composed of collagenous proteins, mainly type II in hyaline cartilage, collagen type X, glycoproteins, proteoglycans, and glycosaminoglycans (Sophia Fox et al. 2009; Yang et al. 2005). The chondrocyte, the only resident cell found in cartilage, proliferates and secretes ECM forming the cartilage template of the skeleton during development. In adults, chondrocytic cells produce and maintain ECM that participates in growth, mechanical support, and function of diarthrodial joints between bones (Sophia Fox et al. 2009; Mackie et al. 2011). Longitudinal bone growth is dependent on the strict temporal regulation and control of chondrocyte proliferation, differentiation, hypertrophy, apoptosis, and vascular invasion within the growth plate by a complex network of regulatory molecules and interactions of cells and ECM (Mackie et al. 2011; Karsenty 2008; Karsenty et al. 2009). Until now, over 400 skeletal disorders have been described, and several are caused by mutations in cartilage ECM genes that are critical to endochondral ossification (Warman et al. 2011). Although individually rare, they cause a significant impact on the quality of life for patients suffering from skeletal abnormalities. Genes encoding cartilage ECM proteins affected by mutations which disrupt growth plate differentiation comprise collagen types II, IX, X, and XI; aggrecan; cartilage oligomeric matrix protein (COMP); and matrilin 3 (Hintze et al. 2008; Chung et al. 2009; Liang et al. 2014; Ho et al. 2007; Mäkitie et al. 2010; Kuivaniemi et al. 1997; Warman et al. 2011; Bateman et al. 2009; Boot-Handford and Briggs 2010; Yang et al. 2005; Briggs et al. 2015). The list of collagenopathies associated with mutations in collagen types II and X is presented in Table 1.

**Table 1:** Skeletal diseases associated with mutations in collagen types II and X

Disease	Locus	Gene	Inh	Protein	MIM	Main clinical features	Ref.
Hypochondrogenesis, achondrogenesis type II (ACG2)	12q13.11	*COL2A1*	AD	Type II collagen	200610	Severe micromelic dwarfism, incomplete ossification of the vertebral bodies, disorganization of the costochondral junction, small chest, prominent abdomen	Nishimura et al. 2005; Forzano et al. 2007; Comstock et al. 2010
Spondyloepiphyseal dysplasia congenita (SEDc, SDC)	12q13.11	*COL2A1*	AD	Type II collagen	183900	Disproportionate short stature (short trunk), abnormal epiphyses, flattened vertebral bodies, myopia, and/or retinal degeneration with retinal detachment and cleft palate	Anderson et al. 1990; Donahue et al. 2003; Nishimura et al. 2005
Kniest dysplasia	12q13.11	*COL2A1*	AD	Type II collagen	156550	Short stature, disproportionate (short trunk), platyspondyly, lumbar kyphoscoliosis, coronal vertebral clefts, hypoplastic pelvic bones, hip dislocation, delayed epiphyseal ossification (early), megaepiphyses (late), narrowing of joint spaces, limited joint mobility, painful joints	Gilbert-Barnes et al. 1996; Nishimura et al. 2005
Stickler syndrome type 1 (arthro-ophthalmopathy)	12q13.11	*COL2A1*	AD	Type II collagen	108300	Clinically and genetically heterogeneous disorder, characterized by ocular, auditory, skeletal, and orofacial abnormalities. Most forms are characterized by the eye findings of high myopia, vitreoretinal degeneration, retinal detachment, and cataracts. Additional findings comprise midline clefting (cleft palate or bifid uvula), Pierre Robin sequence, flat midface, sensorineural or conductive hearing loss, mild spondyloepiphyseal dysplasia, and early-onset OA	Nishimura et al. 2005; Ang et al. 2007; Olavarrieta et al. 2008; Hoornaert et al. 2010
Platyspondylic lethal skeletal dysplasia, Torrance type (PLSD-T)	12q13.11	*COL2A1*	AD	Type II collagen	151210	Decreased ossification of the skull base, disc-like platyspondyly, short thin ribs, hypoplastic pelvis with wide sacrosciatic notches and flat acetabular roof, short tubular long bones with metaphyseal cupping	Nishimura et al. 2004; Zankl et al. 2005
Spondyloepimetaphyseal dysplasia, Strudwick type (SEMD)	12q13.11	*COL2A1*	AD	Type II collagen	184250	Severe dwarfism, superficially resembling the Morquio syndrome, pectus carinatum, and scoliosis which are usually marked. Cleft palate and retinal detachment frequently associated, as in SEDc (183900). A distinctive radiographic feature is irregular sclerotic changes, described as “dappled” in the metaphyses of the long bones	Sulko et al. 2005; Shapiro et al. 2006; Czarny-Ratajczak et al. 2009
Spondyloperipheral dysplasia	12q13.11	*COL2A1*	AD	Type II collagen	271700	Short stature, platyspondyly, mild biconcave disc (fish-mouth vertebrae), kyphosis, short ilia, horizontal acetabulae, flattened capital femoral epiphyses, acetabular spurs (infancy), very short distal phalanges (2nd, 3rd, 4th, 5th), short metacarpals (2nd, 3rd, 4th, 5th), cone-shaped epiphyses, brachydactyly “E-like” changes, short feet, short phalanges, short metatarsals (4th)	Zankl et al. 2004; Bedeschi et al. 2011
Czech dysplasia, spondyloepiphyseal dysplasia with precocious OA	12q13.11	*COL2A1*	AD	Type II collagen	609162	Normal stature, mild platyspondyly, irregular vertebral endplates, narrow intervertebral disc spaces, irregular sclerotic acetabulae, flattened capital femoral epiphyses, narrow iliac wings, narrow short femoral neck, arthralgia, flexion contractures (knee), osteochondromatosis (knee), short metacarpals (4th–5th), onset of joint pain in childhood, waddling gait, hip replacement in early adulthood, hearing loss	Hoornaert et al. 2007; Tzschach et al. 2008; Matsui et al. 2009
Avascular necrosis of the femoral head (ANFH)	12q13.11	*COL2A1*	AD	Type II collagen	608805	Patients present with groin pain, onset of symptoms in 2nd to 5th decades of life, degeneration of hip joint, narrowing of joint space, avascular necrosis/cystic changes/sclerosis of femoral head, generalized osteoporosis (in some patients), mild scoliosis (in some patients)	Liu et al. 2005; Nishimura et al. 2005; Su et al. 2008; Kannu et al. 2011; Li et al. 2014
Legg-Calvé-Perthes disease (LCPD)	12q13.11	*COL2A1*	AD	Type II collagen	150600	Disease onset between 6 and 9 years, short stature, necrosis of capital femoral epiphysis, more severe in females, more frequent in males	Nishimura et al. 2005; Miyamoto et al. 2007; Su et al. 2008; Li et al. 2014
Otospondylomegaepiphyseal dysplasia (OSMED), Nance-Sweeney chondrodysplasia, chondrodystrophy with sensorineural deafness	12q13.11	*COL2A1*	AR	Type II collagen	215150	Short stature, sensorineural hearing loss, epiphyseal dysplasia, premature OA , midface hypoplasia, increased lumbar lordosis, vertebral coronal clefts (newborn), enlarged odontoid (childhood), platyspondyly (childhood), joint contractures and pains, enlarged joints, short hands/ fingers/metacarpals, prominent interphalangeal joints	Miyamoto et al. 2005
Metaphyseal (chondro) dysplasia, Schmid type (MCDS, MCS)	6q21–22.3	*COL10A1*	AD	Type X collagen	156500	Short stature (mild to moderate), adult height 130–160 cm, mild platyspondyly, coxa vara, femoral and tibial bowing, metaphyseal abnormalities of distal and proximal femurs/proximal tibiae and fibulae/distal radius and ulna, metaphyseal cupping of proximal phalanges and metacarpals, waddling gait, leg pain during childhood	Mäkitie et al. 2005; Ho et al. 2007; Mäkitie et al. 2010

AD autosomal dominant; AR, autosomal recessive; COL2A1, collagen type II alpha 1; COL10A1, collagen type X alpha 1; OA, osteoarthritis

## Mutations in collagen type II

Collagen II, a main structural protein of cartilaginous tissues, is first synthesized as procollagen, characterized by the presence of an extended triple-helical domain flanked by the globular N-terminal and C-terminal propeptides (Arnold and Fertala 2013). Biosynthesis of procollagen molecules is a complex process that involves posttranslational modifications of the nascent pro-α chains. These modifications include hydroxylation of proline residues and lysine residues by prolyl-4 hydroxylase (P4H) and lysyl hydroxylase (LH), respectively. In turn, the posttranslational modifications and formation of the triple-helical structure are controlled by molecular chaperones that belong to the group of proteins residing in the ER, which includes heat shock protein 47 (HSP47), Grp78, and protein disulfide isomerase (PDI), amongst which the latter enzyme also acts as the β subunit of the αα/ββ tetramer that constitutes functional P4H (Chung et al. 2009; Ono et al. 2012; Patterson and Dealy 2014). In addition, by binding to the individual pro-α1(II) chains and preventing their premature association, P4H may also act as a chaperone during biosynthesis of procollagen molecules. Procollagen molecules are released from the ER and directed toward secretory pathways following biosynthesis of individual pro-α1(II) chains, their posttranslational modifications, and folding into triple-helical structure (Chung et al. 2009; Ono et al. 2012).

**Fig. 3:**
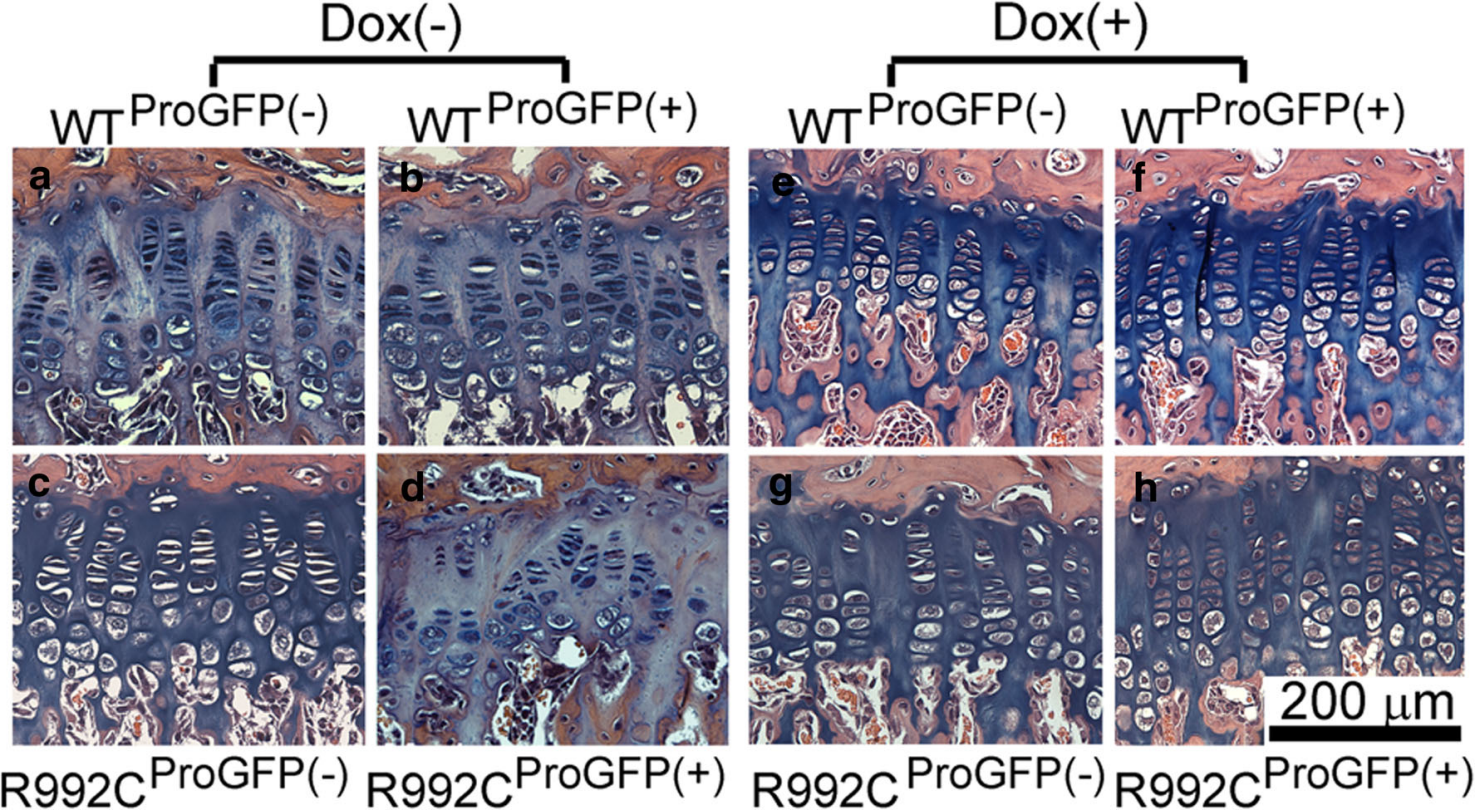
Histology of the tibial growth plates of the 10-week-old WT^ProGFP(−)^ (**a**, **e**), WT^ProGFP(+)^ (**b**, **f**), R992C^ProGFP(−)^ (**c**, **g**), and R992C^ProGFP(+)^ (*D*, *H*) mice maintained in the absence (*A*–*D*) or presence (*E*–*H*) of Dox. In contrast to chondrocytes seen in the growth plates of the 10-week-old WT^ProGFP(−)^ (**a**), WT^ProGFP(+)^ (**b**), and R992C^ProGFP(−)^ (**c**) mice, the columnar organization of chondrocytes in the R992C^ProGFP(+)^ littermates was altered (**d**). For instance, in the R992C^ProGFP(+)^ mice, such alterations were indicated by the presence of *disorganized columns* whose continuity of the typical palisade-like arrangement was often interrupted by extended areas in which the chondrocytes were absent (**d**). Switching off the expression of the R992C ProGFP in the ^Dox^R992C^ProGFP(+)^ mice maintained in Tet-off conditions resulted in developing growth plates in which chondrocytes were organized correctly (**h**). Growth plates from these mice had a normal morphology comparable to that seen in the ^Dox^R992C^ProGFP(−)^ littermates (**g**) as well as their ^Dox^WT^ProGFP(−)^ and ^Dox^WT^ProGFP(+)^ counterparts maintained in Tet-off conditions (**e**, **f**) (reprinted from the *American Journal of Pathology* (Arita et al. 2015) with permission from Elsevier)

To date, about 330 records of mutations in collagen type II α1 (COL2A1) that cause chondrodysplasias have been published (Stenson et al. 2009). These mutations alter the gene encoding the α1 chain of procollagen type II causing various types of the disease, which can be lethal, *i.e.*, hypochondrodysplasia or deforming, *i.e.*, spondyloepiphyseal dysplasia congenita (SEDc), Kniest dysplasias, and Stickler syndrome or may appear as mild knee and hip joint diseases (Nishimura et al. 2005; Su et al. 2008; Warman et al. 2011; Arnold and Fertala 2013; Li et al. 2014). The most frequent amino acid substitutions in collagen type II occur at the G position of the G-X-Y triplets; for instance, a G853E (p.G1053E) substitution was found in a patient with a lethal form of SED. Some reports described substitutions in the Y position; for example, cysteine substitutions for arginine residues were found in the Y positions 75 (p.R275C), 519 (p.R719C), and 789 (p.R989C) in patients with a mild form of SED, a mild form of OA, and a severe form of SEDc (Hoornaert et al. 2006; Hintze et al. 2008). Relatively rare mutations comprise substitutions in the X position of the G-X-Y triplets that were found in patients with Stickler syndrome (Table 1) (Richards et al. 2000). For example, Chung et al. (2009) investigated the R992C (p.R1192C) substitution in the X position of a G-X-Y triplet of collagen type II. Corresponding substitution (p.R1147C) originally described in mice represents skeletal abnormalities similar to those seen in patients with SEDc (Donahue et al. 2003). Using a system expressing recombinant collagen type II, Chung et al. (2009) demonstrated that R992C mutant caused aberrant electrophoretic mobility and was characterized by relatively low thermostability and the presence of intramolecular disulfide bonds. Additionally, the expression of aberrant collagen type II was associated with ER stress and increased apoptosis of cells producing the mutant. Furthermore, Jensen et al. (2011) demonstrated the association between excessive intracellular accumulation of the R992C mutant molecules and ER stress using organotypic cartilage-like constructs. Other substitution in collagen type II (R789C, p.R989C) also caused misfolding of mutant molecules, decreased their thermostabilities, promoted excessive intracellular accumulation, and increased apoptosis (Hintze et al. 2008). These results genuinely have shown that beyond the loss of function effect, certain mutations in collagen type II may also cause a gain-of-function, *i.e.*, cytotoxic effect. Moreover, these data contribute to the understanding of the molecular basis of mutations that trigger pathological changes seen at the level of skeletal tissues and suggest that mutations in collagen type II associated with variations of thermostability and disturbance of correct folding of the collagen triple helix apart from the pathological impact on extracellular collagenous framework also alter intracellular processes. More recently, Arita et al. (2015) employed a SED mouse model to analyze the morphology of the growth plate. This model consists of conditional expression of the R992C mutant or the wild type (WT) transgene by using a tetracycline (Tet)-modulated promoter, leading to the transgene being switched off in the presence of doxycycline (Dox). Morphological analyses of growth plates from newborn, 2-week-old, 6-week-old, and 10-week-old mice suggest excessive accumulation of collagen type II chains within dilated chondrocytes present in growth plates of mice harboring R992C mutants. The presence of similarly dilated cells was neither observed in WT mice nor mice maintained in Tet-off conditions. A histological study of the tibial growth plates showed aberrations in growth plate organization. In contrast to chondrocytes found in growth plates of WT mice, the columnar organization of chondrocytes in mice harboring R992C mutant was disturbed, alterations being indicated by the presence of disorganized columns. Switching off the expression of the R992C mutant in mice with Dox-regulated promoter maintained in Tet-off conditions resulted in developing growth plates with correctly organized chondrocytes (Fig. 3). Abundant collagen type II deposits were noted in the extracellular spaces of growth plates of WT mice, while in contrast, in mice with the mutation, the extracellular content of collagen type II was significantly reduced and its increased intracellular accumulation was clearly visible. Analysis of Sirius red-stained deposits of collagen fibrils present in the growth plates of WT mice indicated their organized pattern of distribution in the longitudinal septa present between adjacent columns of chondrocytes. By contrast, the collagenous matrix present in the growth plates of the mice harboring collagen type II mutant lacked clear structural continuity, and the longitudinal septa were irregularly thickened. In summary, the studies with transgenic mice have shown that the presence of R992C collagen type II results in skeletal aberrations that include alterations of the linear growth. Aberrant columnar organization of chondrocytes was associated with ER stress, abnormal architecture of collagenous matrix, unusual organization of primary cilia, atypical cell polarization, and reduced proliferation of chondrocytes harboring the mutation. In turn, Liang et al. (2014) constructed a mouse model harboring the p.G1170S mutation in collagen type II. Severe defects in skeletal development (chondrodysplasia phenotype) were observed in homozygotes, but not in heterozygotes. Homozygous fetuses were smaller, with shortened and widened long bones, abnormal scapulae, shapeless pelvises, non-ossified middle phalanges, malformed ribcages, and less-mineralized vertebrae. These defects indicate that cartilage shaping was disturbed in mice lacking normal collagen type II and endochondral ossification was slowed. In contrary, skeletons from heterozygotes resembled wild types by appearance. Abnormal features of the skeleton were confirmed at the molecular level as well. Chondrocytes in the resting zones of homozygotes were normally distributed; however, the proliferating zones consisted of fusiform cells decreased in number, aligned transversely and chaotically, while the hypertrophic zone was lost. Proteoglycans, collagen type II, and expression of SOX9 (which regulates the expression of collagen type II) in the growth plate were significantly decreased in homozygotes. The analysis of cultured chondrocytes collected from embryonic cartilage by a confocal microscope showed that mutated type II procollagen in homozygotes assembled into bundles, co-localized with the ER, and was retained intracellularly. Intracellular accumulation resulted in ER distention and ER stress-UPR-apoptosis cascade activation. Additionally, expression of several ER stress-related genes, corresponding to CHOP, total XBP1 (tXBP1), spliced XBP1 (sXBP1), Grp78, ATF4, and ATF6, was upregulated in homozygotes and partly increased in heterozygotes. Cell apoptosis prevented the formation of a hypertrophic zone and disrupted normal chondrogenic signaling pathways. In consequence, in homozygotic mice harboring the p.G1170S mutation, abnormally developed growth plate was found. In contrast, heterozygotes had normal phenotypes with limited ER stress. These results suggest that chondrocytes’ death related to ER stress-UPR-apoptosis cascade was a crucial cause of chondrodysplasia.

A novel COL2A1 missense mutation (p.D1469A) in mice was identified, which was situated in the C-propeptide region of α1 chain (Furuichi et al. 2011). Mutation in this position corresponds to human COL2A1 mutation responsible for platyspondylic lethal skeletal dysplasia, Torrance type (PLSD-T). Skeletal defects found in the homozygotes were similar to those in PLSD-T patients. Accumulation of mutant proteins in abnormally expanded rough ER was accompanied by disrupted secretion of this mutant protein into extracellular space and upregulation of ER stress-related genes, corresponding to Grp78 and CHOP in chondrocytes (Table 2).

**Table 2:** Mutations in *COL2A1* and *COL10A1* genes causing skeletal diseases associated with ER stress

Mutation	Study model	Gene	Protein	Molecular/cellular mechanism	Disease	Ref.
p.R1192C	In vitro, ex vivo, in vivo (mice)	*COL2A1*	Type II collagen	Aberrant electrophoretic mobility and low thermostability, slow rates of secretion into the extracellular space of mutant protein, presence of atypical disulfide bonds, ER stress induction (increased PDI level), increased apoptosis of cells producing mutant collagen (increased amount of cleaved PARP),dilated chondrocytes in tibial growth plate, unusual organization of primary cilia, atypical cell polarization, reduced proliferation of chondrocytes leading to aberrant organization of the growth plate	Spondyloepiphyseal dysplasia (SED)	Chung et al. 2009; Gawron et al. 2010; Jensen et al. 2011; Arita et al. 2015
p.R989C	In vitro	*COL2A1*	Type II collagen	Misfolding, decreased thermostability and excessive intracellular accumulation of mutant molecules, dilated ER cisternae in chondrocytes, increased apoptosis (increased level of cleaved caspase 3)	Severe form of spondyloepiphyseal dysplasia congenita (SEDc,SDC)	Hintze et al. 2008; Jensen et al. 2011
p.G1170S	Ex vivo, in vivo (mice)	*COL2A1*	Type II collagen	Upregulated expression of ER stress-related genes corresponding to CHOP, tXBP1, sXBP1, Grp78, ATF4, ATF6, increased apoptosis (increased level of cleaved caspase 3),dilated ER in chondrocytes with abnormally accumulated mutant collagen and glycogen granules, proliferating zones of the growth plates consisted of fusiform cells decreased in number, chaotically aligned, hypertrophic zone lost	SED	Liang et al. 2014
p.D1469A	In vivo (mice)	*COL2A1*	Type II collagen	Mutated collagen retained in the ER, abnormally expanded ER, upregulated ER stress-associated genes of Grp94 and CHOP in chondrocytes	Platyspondylic lethal skeletal dysplasia, Torrance type (PLSD-T)	Furuichi et al. 2011
p.Y663X p.P620fsX621/c.1859delC FCdel	Human probant, in vivo (mice)	*COL10A1*	Type X collagen (alpha-1 chain)	Growth plate expansion at birth, intracellular retention of mutant collagen type X within the ER of cells in the upper part of the hypertrophic zones, increased levels of Grp78, CHOP, and sXBP1, affected growth plate maturation, impaired longitudinal bone growth	Metaphyseal (chondro) dysplasia, Schmid type (MCDS, MCS)	Ho et al. 2007
p.Y598D, p.G618V, p.N617K, NC1del10	In vitro	*COL10A1*	Type X collagen (alpha-1 chain)	Instability of the mutant transcripts, mutant misfolding leading to formation of aberrant disulfide bonds, retained mutant collagen in the ER, enhanced expression of the sXBP1, and Grp78, UPR activation	MCDS	Wilson et al. 2002; Wilson et al. 2005
p.N617K	In vivo (mice)	*COL10A1*	Type X collagen (alpha-1 chain)	The upper hypertrophic zones of tibial, femur and ribs growth plates markedly expanded (apparently at birth) due to intracellular retention of mutant protein, upregulated gene expression and protein level of Grp78 in the upper hypertrophic zone, significantly induced active ATF6_50_ in hypertrophic chondrocytes expressing mutant collagen type X	MCDS	Rajpar et al. 2009; Wilson et al. 2005
NC1del13	In vivo (mice)		Type X collagen (alpha-1 chain)	Impaired mutant collagen secretion, expansion of hypertrophic zones of growth plates, induction of the sXBP1 and Grp78, upregulated CHOP transcript expression and protein in upper hypertrophic zones	MCDS	Tsang et al. 2007

ER, endoplasmic reticulum; UPR, unfolded protein response; PDI, protein disulfide isomerase; Grp78, glucose-regulated protein 78 kDa; CHOP, CCAAT/enhancer binding protein homologous protein; XBP1, X-box binding protein 1; tXBP1, total XBP1; sXBP1, spliced variant of XBP1; ATF4, activating transcription factor 4; ATF6, activating transcription factor 6; ATF6_50_, active form of ATF6; Grp94, glucose-regulated protein 94 kDa

## Mutations in collagen type X

Collagen type X belongs to non-fibrillar collagens produced by chondrocytes residing within the hypertrophic zone of mammalian growth plates. Each protein chain consists of a carboxyl-terminal non-collagenous domain (NC1), the main triple-helical domain, an amino-terminal non-collagenous domain (NC2), and a signal peptide. Collagen trimers are stabilized and propagate correctly from the C- to the N-terminal end of the molecule as a result of potent hydrophobic interactions between the NC1 domains. Missense, nonsense, and frameshift mutations in collagen type X alpha1 (COL10A1) cause MCDS. Forty of the 42 reported mutations alter the NC1 domain, whereas two alter the signal peptide cleavage site of the collagen type X protein chain. Both, haploinsufficiency and dominant-negative mechanisms have been reported. Affected individuals are clinically normal at birth, but after they start walking, they develop a disproportionate short stature. The shortening and deformities of the long bones are due to impaired function of the thickened and irregular growth plates (Table 1) (Warman et al. 2011; Mäkitie et al. 2005; Bateman et al. 2003, 2005; Chan et al. 1998).

Several reports of in vitro protein assembly assays, analyses of human MCDS cartilage, as well as transgenic mouse models of MCDS provided evidence that COL10A1 mutations inducing MCDS result in some mRNA degradation, but also determine a gain-of-function effect on the growth plate. For instance, MCDS probant heterozygous for a p.Y663X nonsense mutation produced a truncated α1(X) chain lacking the last 18 amino acids of the NC1 domain that did not form stable homotrimers and were unable to form stable heterotrimers with WT α1(X) chains. Iliac crest growth plate cartilage from the probant contained 64 % of WT and 36 % of mutant mRNA, and the hypertrophic zone was disorganized and expanded (Ho et al. 2007). In turn, in transgenic FCdel mice corresponding to a human MCDS, p.P620fsX621 mutation showed an early-onset MCDS phenotype with disproportionate shortening of the limbs and coxa vara deformities of the femoral necks. The severity of changes correlated with higher copy numbers/expression of the transgene and increased levels of sXBP1. The FCdel α1(X) chains that lacked the C-terminal 60 amino acids of the NC1 domain were synthesized by the HC, but the protein accumulated within the ER. Retention of the FCdel α1(X) chains was associated, in a concentration-dependent manner, with increased thickness and abnormal maturation of the hypertrophic zone. Thickening of the hypertrophic zones of the FCdel+/− and FCdel+/+ mice, in the absence of endogenous collagen type X, indicated that the retained protein was accompanied by a gain-of-function effect, activating ER stress signaling and affecting growth plate maturation (Ho et al. 2007). Another group constructed mutants predicted either to prohibit subunit folding and assembly (NC1del10 and p.Y598D, respectively) or to allow trimerization and secretion of mutated α1(X) chains (p.N617K and p.G618V). All four mutations resulted in the formation of aberrant disulfide bonds and significantly increased amounts of BiP, and the sXBP1 mRNA, two key markers of the UPR (Wilson et al. 2002, 2005). In the study of Rajpar et al. (2009), a knock-in mouse model of the COL10A1 (p.N617K) mutation inducing MCDS was generated. It has been demonstrated that the MCDS-associated expanded hypertrophic zone occurred because of disrupted vascular endothelial growth factor (VEGF)- mediated osteoclast erosion of the mineralized cartilage at the vascular invasion front. ER stress and a strong UPR caused by misfolding mutant collagen type X were key features of the hypertrophic chondrocytes in the MCDS mouse. Furthermore, the targeted stimulation of ER stress in hypertrophic chondrocytes in vivo was sufficient to replicate the MCDS growth plate phenotype functionally, demonstrating the central role played by ER stress in the disease mechanism. Similar results were reported in a MCDS-expressing transgenic mouse line harboring a 13-bp deletion in COL10A1 (13del). Mutant collagen type X molecules were accumulated within hypertrophic chondrocytes that underwent ER stress which lead to altered chondrocyte differentiation (Table 2) (Tsang et al. 2007). It is therefore possible, that in vivo interference with the assembly of collagen type X acts dominantly negative, resulting in poor or no secretion of mutant homotrimers and heterotrimers and reduced amounts of normal collagen type X in the ECM. In the absence of mutant collagen type X in the ECM, the phenotype could be attributed to haploinsufficiency for collagen type X (Chan et al. 1998; Bateman et al. 2003). However, if mutant collagen type X is secreted to the ECM, there could be a dominant-negative effect on structural properties of ECM.

## Conclusions and perspectives

The endoplasmic reticulum is one of the major cellular sites for the synthesis, maturation, and folding of secreted, membrane-bound, and organelle-targeted proteins. Perturbations of ER homeostasis via acceleration of unfolded or misfolded proteins cause a stress condition for this organelle. To deal with it and restore a more favorable folding environment, the ER triggers an evolutionarily conserved signaling cascade, named UPR. In addition to physiological processes, increasing evidence suggests that ER stress is involved in certain groups of diseases, for instance neurodegenerative and metabolic diseases as well as in some malignancies, occurring as a result of protein aggregation. Moreover, an increasing number of reports cite ER stress as a novel mechanistic paradigm of connective tissue diseases caused by mutations in several types of collagens. This refers to mutations in collagen type VI that induce mild to severe phenotype of myopathies and mutations in collagen type IV that are responsible for developing of nephropathies, ocular dysgenesis, brain malformations, or cerebral hemorrhages. More importantly, a significant number of reports from in vitro studies and mice models with skeletal abnormalities corresponding to chondrodysplasias point to ER stress as a mechanism involved in the pathogenesis of these group of diseases.

Chondrodysplasias comprise a wide spectrum of phenotypes predominantly affecting cartilage and bone, from the severe disorders that are perinatally lethal to the milder conditions that are recognized in the postnatal period and childhood. The milder chondrodysplasias are characterized by disproportionate short stature, eye abnormalities, cleft palate, and hearing loss. Treatment is difficult and limited, particularly when the pathological process begins before birth and can affect the entire skeletal system. Additionally, considering potential pleiotropic effects of mutant genes, patients suffering from chondrodysplasias may have complications with central and peripheral nervous system, bone marrow, immune system, kidney, heart, etc.

This review focuses on basic mechanisms of ER stress and its significance in certain variants of chondrodysplasia caused by mutations in collagen types II and X. Regarding collagen type II, the following are discussed: substitutions in the X position of a G-X-Y triplet that are present with skeletal abnormalities similar to those seen in patients with SEDc; substitutions in the Y position that lead to abnormalities found in patients with a mild form of SED, a mild form of OA, and a severe form of SED; as well as a missense mutation in the C-propeptide region of α1 chain which is associated with PLSD-T. Considering collagen type X, missense, nonsense, and frameshift mutations responsible for MCDS have been described on the basis of reports from in vitro protein assembly assays, analyses of human cartilage, as well as transgenic mice models. The retained collagen type II/X mutant proteins caused a gain-of-function effect on the growth plate activating ER stress signaling and affecting growth plate organization with decreased thermostability and excessive intracellular accumulation of mutant molecules leading to apoptosis of cells producing collagen mutants as a hallmark. Taking together, efforts should be undertaken in the future investigations to verify the observations from the studies discussed herein, which showed a significant contribution of the ER stress and associated downstream signaling as a pathogenic mechanism of certain variants of chondrodysplasia. A better understanding of this association would be advantageous in designing preventive strategies, early ameliorative management, and/or perhaps novel therapies of the individuals being at risk of developing skeletal diseases. Such interventions potentially translate into a reduction in health costs associated with musculoskeletal disease.

ACG2, achondrogenesis type II; ATF4, activating transcription factor 4; ATF6, activating transcription factor 6; AS, Alport syndrome; AD, autosomal dominant; AR, autosomal recessive; ANFH, avascular necrosis of the femoral head; bZIP transcription factor, basic leucine zipper; BM, Bethlem myopathy; CNX, calnexin; CRT, calreticulin; CREBH, cAMP response element binding protein H; COMP, cartilage oligomeric matrix protein; C/EBP, CCAAT/enhancer binding protein; CHOP, CCAAT/enhancer binding protein homologous protein; COL2A1, collagen type II α1; COL10A1, collagen type X α1; DOX, doxycycline; ER, endoplasmic reticulum; ERAD, endoplasmic reticulum-associated degradation system; ERSE, ER stress response element; eIF2α, eukaryotic translation initiation factor 2α; ECM, extracellular matrix; Grp78, glucose-regulated protein 78 kDa; Grp94, glucose-regulated protein 94 kDa; HSP47, heat shock protein 47; IRE1, inositol-requiring enzyme 1; LCPD, Legg-Calvé-Perthes disease; LH, lysyl hydroxylase; MCDS/MCS, metaphyseal (chondro) dysplasia, Schmid type; OASIS, old astrocyte specifically induced substance; OA, osteoarthritis; OI, osteogenesis imperfecta; OSMED, otospondylomegaepiphyseal dysplasia; PLSD-T, platyspondylic lethal skeletal dysplasia, Torrance type; P4H, prolyl-4 hydroxylase; PDI, protein disulfide isomerase; PERK, protein kinase RNA-like endoplasmic reticulum kinase; PQCS, protein quality control system; RIP-regulated liver-specific transcription factor, regulated intramembrane proteolysis; sXBP1, spliced variant of X-box binding protein 1; SEMD, spondyloepimetaphyseal dysplasia, Strudwick type; SED, spondyloepiphyseal dysplasia; SEDc / SDC, spondyloepiphyseal dysplasia congenita; TBMN, thin-basement-membrane nephropathy; tXBP1, total X-box binding protein 1; UPP, ubiquitin proteasome pathway; UCMD, Ullrich congenital muscular dystrophy; UPR, unfolded protein response; UPRE, UPR element; VEGF, vascular endothelial growth factor; WT, wild type; XBP1, X-box binding protein 1.
